# Measuring knowledge of Alzheimer’s: development and psychometric testing of the UJA Alzheimer’s Care Scale

**DOI:** 10.1186/s12877-019-1086-2

**Published:** 2019-03-04

**Authors:** Laura Parra-Anguita, Inmaculada Sánchez-García, Rafael del Pino-Casado, Pedro L. Pancorbo-Hidalgo

**Affiliations:** Department of Nursing, Faculty of Health Sciences, University of Jaen, Crt. Torrequebradilla s/n. 23071, Jaen, Jaen, Spain

**Keywords:** Alzheimer’s disease, Dementia, Surveys and questionnaires, Nursing homes, Nursing students

## Abstract

**Background:**

Care for people with Alzheimer’s disease or other dementias should be based on up-to-date clinical practice guidelines. The education and training of nurses and other healthcare staff in dementia is a key factor in providing quality care. Knowledge of Alzheimer’s disease can be measured through questionnaires. The aim of this study was to develop and validate a scale to measure Alzheimer’s disease knowledge among both nursing staff and students.

**Methods:**

This was a cross-sectional survey study undertaken in three stages: 1) development of the questionnaire and item wording; 2) content validation by an expert panel; 3) questionnaire testing with two samples to establish psychometric properties. Sample 1 comprised 361 Registered Nurses, Assistant Nurses and eldercare workers from 24 nursing homes in Jaén (southern Spain). Sample 2 comprised 297 nursing students. The data were analysed through item analysis and a Rasch model. Convergent and construct validity and internal consistency were also examined.

**Results:**

The 23-item UJA Alzheimer’s Care Scale shows good outfit and infit values based on the Rasch model. One item presented differential functioning between Registered Nurses and Assistant Nurses. The intraclass correlation coefficient between the UJA Alzheimer’s Care Scale and the Spanish version of the Dementia Knowledge Assessment Tool 2 showed strong agreement among nursing staff (0.63) and students (0.79). The scale is able to distinguish between professionals with low or high knowledge of Alzheimer’s care. The overall Cronbach’s alphas were 0.70 (nursing staff) and 0.82 (nursing students). The intraclass correlation coefficient between the first test and the retest was good (0.84).

**Conclusions:**

The UJA Alzheimer’s Care Scale is a useful tool for measuring knowledge of Alzheimer’s disease and dementia care among nursing professionals or nursing students. The initial validation study obtained good psychometric properties concerning validity and reliability.

**Electronic supplementary material:**

The online version of this article (10.1186/s12877-019-1086-2) contains supplementary material, which is available to authorized users.

## Background

Increased life expectancy and decreasing birth rates are among the causes of population ageing worldwide [1]. It is estimated that between 2015 and 2050, the proportion of the world’s population over 60 years of age will increase from 12 to 22% (from 900 million to about 2000 million people) [2]. Dementia is one of the major health problems affecting older people according to the World Health Organization [2]. Age is the factor responsible for the greatest increase in the risk of dementia, as both the incidence and prevalence of dementia double every 5 years from the age of 65 [3]. Alzheimer’s disease (AD) is the most frequent form of dementia (60–70% of cases), followed by vascular dementia (12.5–25%) [4]. In Spain, the figures of dementia prevalence range from 5 to 14.9% in people aged 65 or older and from 9 to 17.2% in those older than 70 [5, 6].

To address the needs of the ageing population, particularly of people with dementia, the health systems in most countries have had to adapt by modifying socio-economic policies, changing available resources and designing new intervention strategies [2]. The implementation of clinical practice guidelines (CPGs) has a basic role in providing high-quality healthcare for people with dementia. Good CPGs must be developed by multidisciplinary teams and using recommendations based on the best available evidence [7]. However, there is still a gap between knowledge and usual clinical practice. Health professionals know the recommendations for AD care, but frequently do not put them into practice [8, 9]. Specific training for health professionals providing care for AD or those with other forms of dementia is a key factor for quality care in all settings. The literature contains a number of studies measuring the knowledge of health professionals concerning dementia and AD care, using well-validated questionnaires. Education is important as most such studies have concluded that Registered Nurses (RNs) have higher knowledge than Assistant Nurses (ANs) [10, 11]. A wide range of professionals participate in dementia care, with different education and specific training [12]. General practitioners (GPs) have considerable knowledge regarding the diagnosis and treatment of dementia, but relatively low knowledge of epidemiology. These professionals also have difficulties in communicating the dementia diagnosis to patients, coordinating with social services and managing behavioural changes [13]. In nursing homes and other residential facilities, ANs or eldercare staff provide most of the basic care for people with dementia, but these professionals have the lowest levels of training [14]. In some cases, family carers of people with dementia may have greater knowledge regarding care than nurses and other care workers [15]. Some authors highlight that the level of knowledge regarding dementia is not only influenced by training, but also experience and real contact with people with dementia [16]. Low knowledge of AD care can lead to diagnostic mistakes and inadequate treatment, among other issues [12]. Hughes et al. (2008) show that nursing home care staff have little training in the needs of the elderly, especially with regard to mental health, depression and dementia. Improving the training of healthcare staff has a major impact on care. The education of health professionals and specific training are the main factors driving organizational changes and better care. Interactive training and blended learning are the best ways of achieving small but lasting improvements in professional practice [17, 18]. It is also important to consider knowledge of dementia care among healthcare students in the fields of nursing and medicine, inter alia. Several studies have found that clinical placements and mentorship play an important role in improving outcomes [19, 20].

To design appropriate dementia care education programmes for healthcare workers, it is very important to measure the level of knowledge and identify the issues that are not well known. A number of questionnaires and scales have been developed and tested to measure knowledge of dementia and AD. These instruments have different psychometric characteristics and have been validated among different populations [12, 21, 22]. According to the year of publication, some of the most relevant questionnaires are the following: the Alzheimer’s Disease Knowledge Test (ADKT) [23]; the Alzheimer’s Disease Knowledge Scale (ADKS) [11]; the Dementia Knowledge Assessment Tool Version 2 (DKAT2) [24]; the Dementia Knowledge Assessment Scale (DKAS) [10, 16]. All these questionnaires have been developed and tested in English-speaking countries. Searching the literature, we have found no questionnaire on dementia knowledge developed or culturally adapted to Spanish-speaking countries. There are differences in both the culture and health context between Anglo-Saxon countries and Spain and other Spanish-speaking countries. This is the reason that led us to develop and validate a questionnaire specifically targeting Spanish-speaking countries based on recommendations of both international and Spanish guidelines on dementia care. The new developed questionnaire should be focused on nursing care for people with Alzheimer’s disease, covering a range of topics including palliative care. The aim of this study was to develop and test a scale measuring the knowledge about dementia and AD, useful for nursing professionals and nursing students.

## Methods

### Design

The research constituted a cross-sectional survey study to develop and validate a new scale. Both classic measurement theory [25] and item response theory [26, 27] were used for measuring the psychometric properties. The research included three phases, as follows:

1. Development of the questionnaire and item wording.
2. Content validation by an expert panel.
3. Questionnaire testing through a survey in two samples to establish psychometric properties.

The period of data collection was from November 2016 to January 2017.

### Questionnaire development

Before writing the items, a literature search was performed to identify CPGs on dementia and AD care published or updated since 2010. In all, eight CPGs in English or Spanish were recovered. The recommendations included in the CPGs were extracted and grouped by topic and duplications were eliminated. Recommendations (*n* = 566) focused on care or nursing interventions were selected and used to write the items (*n* = 51) of the questionnaire.

### Content validation

A panel of 15 experts in dementia care (5 nurses working in universities, 9 clinical nurses working in nursing homes or in dementia care specialised centres and 1 general practitioner) was used for content validation. All of them have more than 10 years of professional experience. Each expert rated the relevance of the items with a 5 point scale (1 No relevant to 5 Very relevant). The V- Aiken index [28] was calculated to establish the consensus among the experts, using the value of 0.80 as threshold for retaining the items. Three consecutive rounds were conducted, first round yielded 48 recommendations selected from which 51 items were written for the questionnaire; second round yielded 22 items accepted and 10 items were modified and moved on to the third round. After the whole process, we produced an initial version of the scale with 30 items. Upon attaining expert consensus, the questionnaire was tested for comprehension among a small sample of health professionals.

### Psychometric testing: Sampling

The questionnaire was tested among two different samples. Sample 1 comprised nursing professionals, i.e. Registered Nurses ([RNs], 4-year university degree), Assistant Nurses ([ANs], 2-year diploma) and eldercare workers (1-year technical education), working in 24 nursing homes in the province of Jaen (southern Spain). Sample 2 comprised nursing students (2nd, 3rd and 4th year) from the Faculty of Health Sciences of the University of Jaen (Spain). The sample size in each case was estimated at 300 (for the 30-item version of the questionnaire), according to the methodological recommendations for the validation of questionnaires, namely 5–10 individuals per item [29].

### Instruments

Three instruments were used to collect the data: a questionnaire gathering participants’ demographic data; a questionnaire concerning knowledge of care for people with AD and other dementias (newly developed); the Spanish version of the DKAT2.

#### Questionnaire concerning knowledge of care for people with Alzheimer’s disease and other dementias

The initial 30-item version of the questionnaire obtained after content validation and pilot testing was used. For each item there were three response options: “Yes”, “No” and “I don’t know”. For some items “Yes” was the correct response and for others it was “No”. The “I don’t know” option was included to allow respondents to acknowledge ignorance. The questionnaire was self-administered.

#### Dementia knowledge assessment tool 2 (DKAT2)

A Spanish version of the DKAT2 questionnaire was used to test the convergent validity of the new questionnaire. The original DKAT2 instrument, with 21 items, has good internal consistency (Cronbach’s alpha = 0.79) and three response options: “Yes”, “No” and “I don’t know”. The questionnaire was developed and tested in Australia [24]. Permission to use and translate this questionnaire was obtained from the authors. The Spanish-adapted version was developed by translation and back-translation undertaken by two independent translators; the back-translated version was revised by one of the authors of the original tool (Christine Toye). The Spanish version of DKAT2 has adequate internal consistency (nursing professionals: Cronbach’s alpha = 0.76; students: Cronbach’s alpha = 0.83).

### Data collection procedure

To access nursing home staff, the directors of all nursing homes in the province of Jaen (public and private management) were contacted and asked to participate in the study. Only two centres refused to participate. After approval, each centre received the appropriate number of questionnaires according to the staff numbers. Completed questionnaires were collected over approximately two weeks. A small sub-sample of nursing home staff completed the questionnaires twice (over a three-week interval) in order to do a test-retest. For nursing students, lecturers were informed of the aims of the study and approval was obtained to administer the questionnaires to students in the classroom.

### Ethics

The Committee of Research Ethics of Jaen approved the study. The anonymity and confidentiality of the data were guaranteed in accordance with the Spanish Law of Data Protection.

### Data analysis

Data were tabulated, coded and cleaned in a spreadsheet before the analysis. For each scale, the total score was calculated by adding the number of items with the correct answer. Several methods were used for the analysis, namely item analysis, Rasch modelling and testing validity and reliability.

#### Item analysis

The analysis was conducted for the two samples, but the values obtained from the sample of nursing professionals were used to make decisions concerning the retention or elimination of items. Three indices were calculated for each item: the difficulty index (DF: percentage of correct answers), the ignorance index (percentage of “I don’t know” answers) and the discrimination index (difference between the percentage of correct answers of the 27% of the questionnaires with the highest overall score minus the 27% of the questionnaires with the lowest overall score) [29, 30]. Items with a discrimination index lower than 10% were eliminated for analysis, as these items were not useful for distinguishing between participants with a high or low knowledge. According to the DF, items were classified into six categories: very easy (> 90% correct answers); easy (75.1–90%); somewhat easy (50.1–75%); somewhat difficult (25.1–50%); difficult (10.1–25%) and very difficult (< 10%).

#### Rasch model

The version of the questionnaire produced after the item analysis was further analysed using a Rasch model. The Rasch model is a method based on item response theory, undertaken for the psychometric validation of questionnaires; it allows differentiation between information concerning the items and persons’ performance. Rasch analysis was conducted in JMetrik software using the joint maximum likelihood method [31]. Fit statistics were calculated: unweighted mean square of standardized residuals (outfit) and weighted mean square of standardized residuals (infit). The value 1 denotes a perfect fit; outfit and infit values between 0.5 and 1.5 were deemed acceptable [32]. The statistic Yen’s Q3 was used to test the local independence between items [33]. Finally, we conducted two differential item functioning (DIF) analyses, the first between nursing professionals and nursing students and the second between RNs and ANs. Effect sizes (common Odd Ratio) and 95% confidence intervals were estimated, considering that an item has no DIF when the common OR is between 0.65 and 1.53; has a large DIF when common OR < 0.53 or > 1.89; and has a low DIF when common OR range between 0.53–0.65 or 1.53–1.89 [34].

#### Validity

We tested both convergent criterion validity and construct validity. For convergent criterion validity we compared the newly developed questionnaire with the DKAT2 questionnaire in the Spanish version (as the gold standard). The intraclass correlation coefficient (ICC) was used as the statistic for concordance estimation. Also, we used the Bland–Altman graphic method, which shows the plot of difference between the two measurements versus the average of the two scores [35]. Construct validity was tested through exploratory factorial analysis (EFA) with principal axis factorization as the extraction method and different rotations (varimax, quartimax and oblimin). The method of Parallel analysis with Horn’s criteria was used to calculate the number of factors to extract in the EFA [36, 37]. Each model was tested against the theoretical model that has guided the construction of the questionnaire.

Furthermore, we tested a hypothesis in known groups (groups with expected high and low knowledge of dementia care), i.e.:

- Nursing professionals versus students.

- RNs versus ANs or eldercare workers.

- Staff who had attended specific courses versus those who had not.

- Students with some care experience versus those without.

The adjustment of the knowledge score data to the normal distribution was tested. Comparisons of mean knowledge scores were undertaken using Student’s t test or one-way ANOVA.

#### Reliability

The internal consistency of the questionnaire was estimated using Cronbach’s alpha and the ICC statistics. Temporal stability (test-retest) was measured using the ICC between the first and second administration of the questionnaire.

## Results

### Characteristics of participants

Sample 1 comprised 361 members of nursing staff from the 24 nursing homes (response rate 51.5%). Staff included RNs, ANs and eldercare workers. Sample 2 comprised 297 students enrolled in the 2nd, 3rd and 4th years of a nursing degree in the Faculty of Health Sciences at the University of Jaen (response rate 67.3%). Table 1 shows the main characteristics of these samples.

**Table 1:** Sociodemographic characteristics of the samples

Variables	Nursing home staff *N* = 361 Frequency (%)	Nursing students *N* = 297 Frequency (%)
Age (Mean [SD])	37.71 [1.26]	21.82 [4.62]
Gender		
Female	342 (94.7%)	237 (79.8%)
Male	19 (5.3%)	57 (19.4%)
Professional category		
Registered Nurses	69 (19.1%)	
Assistant Nurses	242 (67.0%)	
Eldercare workers	50 (13.9%)	
Years of experience		
< 5	136 (37.8%)	
5–15	175 (48.6%)	
> 15	49 (13.6%)	
Have attended training courses on AD		
Yes	288 (79.8%)	
No	72 (19.9%)	
Year on nursing degree		
2nd		119 (40.2%)
3th		84 (28.3%)
4th		93 (31.3%)
Experience caring for a family member with AD		
Yes		98 (33.0%)
No		199 (67.0%)

*AD* Alzheimer’s disease, *CPG* clinical practice guideline

### Item analysis

Difficulty, ignorance and discrimination indices were estimated from the 30-item version of the questionnaire. According to the percentage of correct answers (difficulty index) there were 13 items that were very easy, 5 items that were easy, 8 items that were somewhat easy and 4 items that were somewhat difficult. No items were difficult or very difficult. Six items presented a discrimination index lower than 10%, so they were removed. An additional item had a discrimination index of 15.6%, but included more than one concept, so it was also removed. This 23-item version of the questionnaire was used for analysis and named the UJA Alzheimer’s Care Scale (Table 2).

**Table 2:** The UJA Alzheimer’s Care Scale: Final 23-item versions (Spanish and English) with the correct answer in capitals

Versión en español	English version
A continuación, hay una serie de recomendaciones de cuidados para las personas con enfermedad de Alzheimer y otras demencias, algunas son correctas y otras incorrectas. Por favor, lea cada recomendación cuidadosamente, y marque el recuadro correspondiente Sí o No, **según considere que es o no una recomendación correcta, según las guías de práctica clínica actuales**. Si cree que no conoce la respuesta marque No sé. Intente no dejar ninguna en blanco.	Next, there are some recommendations regarding care for people with Alzheimer’s disease and other dementias, some of which are correct and others incorrect. Please read each recommendation carefully and tick one box, “Yes” or “No”, **to indicate whether you consider it correct or not according to current clinical practice guidelines**. If you don’t know, please tick “I don’t know”. Try not to leave any blank boxes.
**Opciones de respuesta:** **Si/No/No se**	**Response options:** **Yes/No/I don’t know**
1. En caso necesario, la contención mecánica se puede utilizar como sustituto de vigilancia o por conveniencia de los profesionales. (FALSO)	1. If needed, mechanical restraints can be used as a substitute for surveillance or for the convenience of professionals. (FALSE)
2. Usar la escala de Zarit para la cuantificación de la carga del cuidador. (CIERTO)	2. The Zarit scale is used to quantify the caregiver’s burden. (TRUE)
3. Cuando la familia no puede garantizar la atención a la persona con demencia, su ingreso en una institución evita el aislamiento social y previene el maltrato. (CIERTO)	3. When families cannot guarantee care for people with dementia, admission to a facility may avoid social isolation and prevent abuse. (TRUE)
4. Mantener la dieta normal, mientras se está evaluando la causa de disfagia. (FALSO)	4. Provide a normal diet, while assessing the causes of dysphagia. (FALSE)
5. Asociar medidas no farmacológicas y farmacológicas para el manejo de los diferentes síntomas conductuales y psicológicos de la demencia. (CIERTO)	5. Non-pharmacological and pharmacological measures should be used together to manage the different behavioural and psychological symptoms of dementia. (TRUE)
6. Notificar la existencia de maltrato o su sospecha, no corresponde a profesionales de enfermería, sino a otros profesionales. (FALSO)	6. Reporting the existence or suspicion of abuse is not a matter for nurses or elderly care workers, but for other professionals. (FALSE)
7. La gestión de la agitación extrema, violencia y agresividad debe tener lugar en un ambiente seguro, de baja estimulación, separados de otros usuarios del servicio. (CIERTO)	7. The management of extreme agitation, violence and aggressiveness must take place in a safe, low-stimulation environment, separate from other users of the service. (TRUE)
8. La primera línea de tratamiento para los trastornos psicológicos y del comportamiento es farmacológica. (FALSO)	8. Specific drugs are the first option for treatment of psychological and behavioural disorders. (FALSE)
9. Registrar en la historia clínica la medida de contención mecánica, tipo y fecha de aplicación, motivo, pauta de cuidados y el consentimiento informado. (CIERTO)	9. The application of mechanical restraints, type and date of application, reason, care provided and informed consent should be recorded in the Patient Medical Record. (TRUE)
10. Los cuidados paliativos han de integrar aspectos psicosociales, espirituales, culturales y de apoyo a los familiares. (CIERTO)	10. Palliative care must include psychosocial, spiritual, cultural and family support aspects. (TRUE)
11. Realizar programas de actividad física a largo plazo para mantener la funcionalidad de los pacientes con demencia institucionalizados. (CIERTO)	11. Conduct long-term physical activity programs to maintain the functional capacity of institutionalized dementia patients. (TRUE)
12. Utilizar la vía oral para el aporte de líquidos en la etapa final de la vida, siempre que sea posible. (CIERTO)	12. Use the oral route for fluid supply at the end of life, whenever possible. (TRUE)
13. Informar a la familia y a los cuidadores de la situación de muerte cercana no mejora la atención en los últimos días. (FALSO)	13. Informing family and caregivers of the near death status does not improve care in the last few days. (FALSE)
14. Informar al cuidador sobre la enfermedad y sus posibles complicaciones, y los recursos sociales y sistemas de apoyo. (CIERTO)	14. Inform the caregiver about the disease and its possible complications, and the social resources and support systems available. (TRUE)
15. La modificación de la conducta, la higiene programada y la micción inducida aumentan la incontinencia urinaria en sujetos con demencia. (FALSO)	15. Behaviour modification programmed hygiene and induced micturition increase urinary incontinence in patients with dementia. (FALSE)
16. Conocer quién es el representante del paciente para incluirlo en la toma de decisiones y en la planificación de cuidados. (CIERTO)	16. Identify who is the patient’s representative to include him or her in decision-making and care planning. (TRUE)
17. Informar y formar a los cuidadores para prevenir la aparición de los síntomas conductuales y psicológicos de la demencia. (CIERTO)	17. Caregivers should be informed and trained to prevent the onset of behavioural and psychological symptoms of dementia. (TRUE)
18. Aconsejar a la persona con demencia, que realice el documento de voluntad vital anticipada en etapas tempranas de la enfermedad. (CIERTO)	18. Advise the person with dementia, to prepare a living will document in the early stages of the disease. (TRUE)
19. Proporcionar atención integral al cuidador, incluyendo asesoramiento y soporte emocional. (CIERTO)	19. Provide comprehensive care to the caregiver, including counselling and emotional support. (TRUE)
20. Los programas de intervención sobre las actividades de la vida diaria no disminuyen la sobrecarga del cuidador a medio plazo. (FALSO)	20. Intervention programmes in activities of daily living do not reduce the caregiver burden in the medium term. (FALSE)
21. Registrar en la historia clínica datos sobre forma de inicio, progresión, síntomas psicológicos y del comportamiento. (CIERTO)	21. Record in the Patient Medical Record data on the form of onset, progression, and psychological and behavioural symptoms. (TRUE)
22. Los planes de cuidados deben abordar las actividades de la vida diaria para maximizar la actividad independiente, mantener la función, adaptar y desarrollar habilidades. (CIERTO)	22. Care plans should address activities of daily living to maximize independent activity, maintain function and adapt and develop skills. (TRUE)
23. Utilizar sonda nasogástrica o gastrostomía percutánea en el paciente con demencia avanzada como vía rutinaria de alimentación, si disfagia. (FALSO)	23. Use nasogastric tube or percutaneous gastrostomy in the patient with advanced dementia as a regular feeding route, if dysphagia. (FALSE)

### Rasch model

Table 3 shows the fit values for the 23-item UJA Alzheimer’s Care Scale (outfit and infit). All the items have very good fit (WMS and UWMS values lower than 1.20). The difficulty values range from − 2.12 for the easiest item (item 14: “Inform the caregiver about the disease and its possible complications, and the social resources and support systems available”) to 2.43 for the most difficult (item 23: “Use a nasogastric tube or percutaneous gastrostomy in the patient with advance dementia as a regular feeding route if dysphagia”).

**Table 3:** Rasch model for the 23-item UJA Alzheimer’s care scale

Items	Difficulty (Standard error)	WMS (infit)	UMS (outfit)
1	0.84 (0.09)	1.07	1.12
2	1.36 (0.09)	1.04	1.02
3	0.54 (0.09)	1.12	1.22
4	0.94 (0.09)	0.97	0.98
5	−0.62 (0.11)	1.00	0.88
6	0.02 (0.10)	1.08	1.03
7	0.28 (0.09)	1.02	1.01
8	1.42 (0.09)	1.07	1.20
9	−1.29 (0.14)	0.90	0.70
10	1.35 (0.14)	0.90	0.79
11	−0.61 (0.11)	0.99	1.25
12	0.47 (0.09)	1.05	1.09
13	1.41 (0.09)	0.99	1.00
14	−2.12 (0.19)	1.00	0.92
15	1.83 (0.09)	0.96	1.02
16	−1.73 (0.17)	0.95	0.74
17	−1.13 (0.13)	0.99	1.10
18	0.88 (0.09)	1.06	1.02
19	−1.65 (0.16)	0.95	0.95
20	1.22 (0.09)	0.94	0.90
21	−1.74 (0.17)	0.95	0.68
22	−1.39 (0.15)	0.90	0.72
23	2.43 (0.10)	1.01	1.12

Estimated using the joint maximum likelihood method. WMS: weighted mean square of standardized residuals (infit). UMS: unweighted mean square of standardized residuals (outfit). Values from 0.80 to 1.20 indicate a good fit to the model

Two analyses of differential item functioning were conducted, one between professionals and students and the other between ANs and RNs. This analysis establishes if all the items perform equally well in the different populations in which the questionnaire is intended to be used. The results are shown in Table 4. Between RNs and ANs only one item (item 2- Use the Zarit scale to quantify the caregiver’s burden) has a significant amount of DIF, favouring the RN group. Between nursing professionals and students there are five items with DIF. Two of these favour the group of students: item 2-Use the Zarit scale to quantify the caregiver’s burden) and item 5-Associate non-pharmacological and pharmacological measures to manage the different behavioural and psychological symptoms of dementia. Three favour the group of nursing staff: item 4- Provide a normal diet while assessing the causes of dysphagia; item 7-The management of extreme agitation, violence and aggressiveness must take place in a safe, low stimulation environment, separate from other users of the service; item 12-Use the oral route for fluid supply at the end of life, whenever possible. Because the amount of DIF was low in these 6 items, we decided to retain them in the questionnaire.

**Table 4:** Analysis of differential item functioning for the 23-item UJA Alzheimer’s care scale

	Nursing students/Nursing staff (reference group) *N* = 653		RNs/ANs and eldercare workers (reference group) *N* = 283	
Item	OR (95% CI)	DIF*	OR (95% CI)	DIF
1	1.26 (0.89–1.76)		1.26 (0.62–2.56)	
2	**0.25 (0.17–0.36)**	+	**0.23 (0.11–0.46)**	+
3	1.39 (0.99–1.95)		1.96 (0.98–3.93)	
4	**2.52 (1.77–3.59)**	–	1.97 (0.85–4.55)	
5	**0.39 (0.24–0.62)**	+	0.49 (0.13–1.84)	
6	0.57 (0.36–0.85)		0.27 (0.08–0.86)	
7	**2.23 (1.54–3.22)**	–	1.31 (0.55–3.11)	
8	0.54 (0.38–0.77)		1.64 (0.82–3.27)	
9	2.29 (1.21–4.33)		0.00 (0–0)	
10	0.52 (0.28–0.98)		0.29 (0.04–2.11)	
11	1.40 (0.89–2.22)		1.19 (0.48–2.98)	
12	**3.95 (2.72**–**5.72)**	–	2.89 (1.28–6.57)	
13	1.38 (0.97–1.96)		1.26 (0.65–2.45)	
14	0.34 (0.15–0.78)		0.00 (0–0)	
15	2.13 (1.47–3.08)		0.61 (0.30–1.23)	
16	0.58 (0.29–1.14)		0.41 (0.02–7.44)	
17	0.74 (0.43–1.26)		2.34 (0.85–6.40)	
18	0.59 (0.42–0.84)		0.87 (0.45–1.68)	
19	0.68 (0.35–1.31)		2.19 (0.55–8.72)	
20	0.87 (0.60–1.24)		1.09 (0.55–2.16)	
21	0.40 (0.20–0.81)		2.07 (0.36–11.74)	
22	1.47 (0.79–2.76)		2.45 (0.48–12.56)	
23	1.22 (0.83–1.79)		0.72 (0.39–1.35)	

*DIF: Evidence for differential item functioning, items with a significant amount of DIF in bold

+ Favouring the focal group. - Favouring the reference group

*RNs*: Registered Nurses, *ANs* Assistant Nurses

### Convergent criterion validity

The concordance of the scores obtained with the UJA Alzheimer’s Care Scale and the DKAT2 Spanish version is shown in Table 5. The ICC ranges from 0.56 in the AN group to 0.79 for the student group.

**Table 5:** Convergent validity between the UJA Alzheimer’s Care Scale and the Dementia Knowledge Assessment Tool (DKAT2) (Spanish version)

Groups for comparison	*N*	ICC (95% CI)
Nursing staff (nursing homes)		
All	350	0.63 (0.54–0.69)
Registered Nurses	68	0.68 (0.48–0.80)
Assistant Nurses	233	0.56 (0.43–0.66)
Eldercare workers	49	0.69 (0.46–0.83)
Nursing students	274	0.79 (0.73–0.83)

ICC = intraclass correlation coefficient

95% CI = 95% confidence interval

**Fig. 1:**
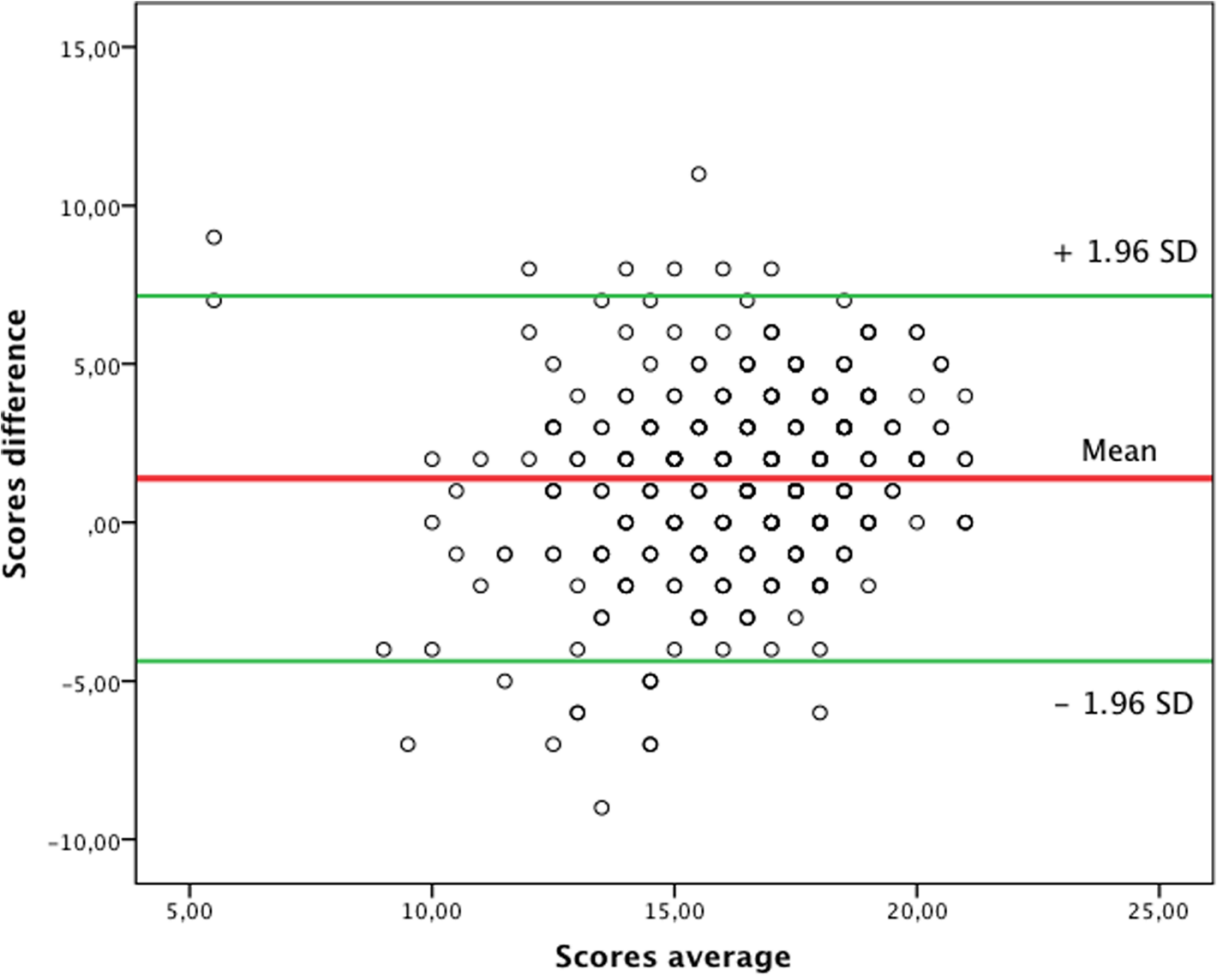
Concordance between the UJA Alzheimer’s Care scale and the DKAT2 scale measured through the Bland-Altman graphic method, for the nursing staff group. The plot shows the difference between the scores on both scales (Y axis) and the average of the scores (X axis)

The Bland–Altman plot also show good concordance, with most of the values within the 95% confidence interval (Fig. 1).

### Construct validity

Parallel analysis initially yielded three factors to extract. We tested different exploratory factor analysis models with main axis factorization extraction using one to four factors and several rotation methods (varimax, quartimax and oblimin). None of these models with more than one factor showed high values of explained variance, nor did they exhibit an adequate fit to a theoretical model of the latent variable measured by this questionnaire. Thus, the UJA Alzheimer’s Care Scale is proposed as a one-dimensional scale.

We hypothesized that the scores obtained with the UJA Alzheimer’s Care Scale would be higher in some groups with a priori greater knowledge of AD. Score data were normally distributed, so parametric tests were used. The test for means comparisons shown in Table 6 confirm this hypothesis. The scale is able to differentiate correctly between people with a high and low knowledge.

**Table 6:** Construct validity of the UJA Alzheimer’s Care Scale, comparing known groups with high or low expected level of dementia care knowledge

Groups for comparison	Score in the scale Mean (Std Dev)	Statistic*	*p*-value
Category			
Nursing home staff (*N* = 360)	16.94 (3.03)	t = 6.17	*p* < 0.0001
Nursing students (*N* = 291)	15.37 (3.45)		
Education			
Registered Nurses (*N* = 69)	19.16 (2.48)	F = 26.02	*p* < 0.0001
Assistant Nurses (*N* = 241)	16.46 (2.98)		
Eldercare workers (*N* = 50)	16.24 (2.57)		
Have attended some training course			
Yes (*N* = 288)	17.23 (2.98)	t = 3.17	*p* < 0.0001
No (*N* = 72)	15.81 (3.00)		
Students with experience in caring for family members with dementia			
Yes (*N* = 96)	16.08 (3.27)	t = 2.45	*p* = 0.015
No (*N* = 193)	15.03 (3.50)		

*t* = Student’s t-test, *F* = One-way analysis of variance

### Reliability

The internal consistency of the 23-item UJA Alzheimer’s Care Scale was Cronbach’s alpha = 0.70 for nursing staff and 0.82 for students. There was no item that could increase the value of Cronbach’s alpha if deleted.

### Temporal stability

A sub-sample of 21 nursing staff members from nursing homes completed the scale twice over a three-week interval (test-retest). The ICC between the two administrations was 0.84 (95% CI = 0.60–0.93).

## Discussion

This research has focused on the development and validation of a new tool to measure knowledge of care for people with AD and dementia in Spain, that could be used in or easily adapted to other Spanish-speaking context. The UJA Alzheimer’s Care Scale comprises 23 items based on recommendations extracted from current CPGs on AD care and treatment. This scale could be used by a range of nursing staff, from RNs to ANs and to eldercare workers, as well as by nursing students. It is not intended for use in GPs because the scale is focused on nursing care, but not in treatment or diagnosis topics. The scale has good content validity based on the wording of items from clinical recommendations and a robust process of review by a panel of experts. The UJA Alzheimer’s Care Scale shows adequate psychometric values for validity and reliability. It is a scale easy to administer and can usually be completed in 5–10 min. The scale could be obtained through the project web site [38] also as Additional file 1.

It is important to highlight that this scale has three response options, including the “I don’t know” option. Several authors have stated that having three response options is better than two options (Yes, No) in knowledge scales because respondents can acknowledge their ignorance concerning a specific theme, avoiding the need to mark “Yes” or “No” at random [39, 40]. Our results concur with those of other studies in that internal consistency increases with the inclusion of the third response option in the knowledge scale [41]. This feature of the scale allows the identification of those topics on which staff or students have erroneous knowledge (errors) vs topics of which respondents recognize having no knowledge. We think that this is useful information when planning educational activities.

The newly developed UJA Alzheimer’s Care Scale contains items exploring person-centred care, like the DKAS [10], as opposed to other scales focusing predominantly on biomedical issues [11, 21, 23, 42].

Our scale has a robust building process, starting from CPGs and including the collaboration of a large panel of 15 experts. Having a panel with a sufficient number of experts is an important point for content validation when developing scales. Most of the previously developed AD knowledge questionnaires have had between 4 and 10 experts [24, 42–44] or the authors acting as experts [11]. Only the DKAS, with 18 experts, had a higher number than ours [10].

Our purpose was to develop a final version of this scale able to measure the latent variable “Knowledge of AD and dementia care” among a wide range of nursing professionals, so we needed to include items with different levels of difficulty. Through the item analysis, some of the easy or very easy items were deleted because they made no contribution to the discriminant capacity of the scale. Analysis with the Rasch model confirmed that the 23-item final version has a good fit and has items with different levels of difficulty. The scale works well with both nursing staff from nursing homes and nursing students, although there are five items that present differential functioning in professionals and in student, namely: the caregiver burden; nutritional care; behavioural symptom management; agitation and aggressiveness management.

The UJA Alzheimer’s Care Scale is a one-dimensional scale that produces a total score to measure the amount of knowledge concerning AD, just by adding up the number of correct answers. Although the process of writing the items started by grouping them into several categories according to the CPG recommendations (such as Basic care; Caregivers; Diagnosis; Behavioural symptoms; End of life decisions), when the scale is used by nursing staff or students, there are no data to support an internal structure with sub-scales or factors. We consider that this one-dimensional structure is an advantage in its use, because it has just one score and is easy to use. Also, it is comparable with studies that have used most dementia knowledge tools, such as DKAT2 [24] or ADKS [11].

Overall, the new scale has good evidence of validity. Convergent validity is strong when it is compared with the DKAT2 scale, which has been well validated and widely used in recent research [15, 24, 45]. Our research also provides evidence of construct validity for the UJA Alzheimer’s Care Scale. This scale is able to discriminate between people with different levels of knowledge [21]. Our data agree with some other validation studies for the ADKS [11], KAML-C [43], UAB-ADKT [21], DK-20 [44], DKAT2 [24] and DKAS [10].

This new scale also has evidence of reliability, high internal consistency with values similar to other scales for nurses, DKAT2 [24]; ADKS [11]; AUB-ADKT [21]; also for nursing students, DQ [42], DKAS [10] and KAML-C [43]. The measures of AD knowledge produced by this new scale appear to be stable over time. Only a few validation studies have conducted a test-retest – ADKS [11], AUB-ADKT [21] and DK-20 [44] – but our data are consistent with most of them. Overall, our research provides data to support the reliability of the UJA Alzheimer’s Care Scale for use by both nursing staff and students.

### Limitations and strengths

This research has several limitations that should be considered. Firstly, the sampling was not random and some nursing homes had a low response rate. A disproportionate number of staff with more knowledge or motivation may have completed the survey, so this could lead to an overestimation in the results for AD knowledge. Second, the sample of nursing students was obtained from only one centre, so it is not possible to generalize these results.

As strengths, we can mention the rigorous and robust process in the development and content validation of this scale. Also, we would highlight the large number of nursing homes (up to 24) that participated in the study. There were centres with different characteristics in terms of size, location and type of management. Being a multi-centre study increases the representativeness of the results.

The UJA Alzheimer’s Care Scale has shown sufficient evidence of validity and reliability in this initial validation study to warrant further research on its use. For future studies, it will be necessary to test this scale with other samples of nurses working in hospitals or primary care. In addition, it should be used with a large sample of nursing students from different universities to check its performance. Finally, we think that the scale could be used with family caregivers who care for people with dementia as a way of identifying their formative needs.

## Conclusions

The UJA Alzheimer’s Care Scale is a tool useful for measuring knowledge of AD or dementia care among nursing professionals and nursing students in Spain and useful for other Spanish-speaking countries. The English version of this scale should be tested in samples of English-spoken nurses and students. It is a self-administered scale, easy to use in both the paper and electronic versions. The initial validation study has obtained good psychometric properties for validity and reliability. This scale could be used in research as a tool to measure knowledge in intervention studies or in education for nursing home staff or nurses in general and also as a tool to identify areas that need improvement and to plan training activities.

## Additional file

Additional file 1: UJA Alzheimer’s Care Scale. Full text of the questionnaire developed. (PDF 293 kb)

## Abbreviations

AD: Alzheimer’s disease
ADKS: Alzheimer’s Disease Knowledge Scale
ADKT: Alzheimer’s Disease Knowledge Test
CPG: Clinical Practice Guideline
DK-20: Dementia Knowledge 20
DKAS: Dementia Knowledge Assessment Scale
DKAT2: Dementia Knowledge Assessment Tool Version 2
DQ: Dementia Quiz
ICC: intraclass correlation coefficient
UAB-ADKT: University of Alabama Alzheimer’s Disease Knowledge Test
