# Supplementary material for: Measuring knowledge of Alzheimer’s: development and psychometric testing of the UJA Alzheimer’s Care Scale
Source: BMC Geriatr. 2019 Mar 4;19:63. doi: 10.1186/s12877-019-1086-2 (PMC6398252; doi:10.1186/s12877-019-1086-2)
Supplement: Supplementary file 1 — UJA Alzheimer’s Care Scale. Full text of the questionnaire developed. (PDF 293 kb) [file 12877_2019_1086_MOESM1_ESM.pdf]

UJA ALZHEIMER´S CARE Scale Knowledge on Alzheimer's disease Care Scale

This scale could be used for research or clinical purposes provided the source is cited. If you use or modify this scale, please report to authors by –email to: lparra@ujaen.es

Cite as: Parra-Anguita L., Sanchez-Garcia, I., Del-Pino-Casado, R., Pancorbo-Hidalgo P.L. Measuring knowledge of Alzheimer's: development and psychometric testing of the UJA Alzheimer's Care Scale. BMC Geriatrics. 2019. In press.

Available at: http://cuidsalud.com/en/inv/knowledge-alzheimer/

This scale measures the overall knowledge on key recommendations to care for people with Alzheimer's disease or other dementias. It could be used for nursing staff (Registered nurses, Assistant nurses of Eldercare workers).

SCORING INSTRUCTIONS

Overall knowledge score.

To calculate this score, 1 point is added for each item with a correct answer.

- YES. Items 2, 3, 5, 7, 9, 10, 11, 12, 14, 16, 17, 18, 19, 21 and 22 add 1 point for each answer YES.
- NO. Items 1, 4, 6, 8, 13, 15, 20 and 23 add 1 point for each answer NO..

Items answered with "Don't know" are scored with 0 points These items can be taken into account to identify areas of ignorance.

The maximum score is 23 points (Knowledge index 100%). Several indices can be calculated from the overall score:

- Knowledge index: Overall score / 23 X 100
- Ignorance index: Number of Don't know answers / 23 X 100

UJA ALZHEIMER´S CARE Scale (English version)

GENERAL INSTRUCTIONS

Next, there are a series of care recommendations for people with Alzheimer's disease and other dementias, some of which are correct and some of which are incorrect.

Please read each recommendation carefully and tick one box, “Yes” or “No”, to indicate whether you consider it correct or not according to current clinical practice guidelines. If you don´t know, please tick “I don´t know”. Try not to leave any blank boxes.

	Yes	No	Don’t know
1- If needed, mechanical restraints can be used as a substitute for surveillance or for the convenience of professionals.	○	○	○
2- The Zarit Scale is used to quantify the caregiver`s burden.	○	○	○
3- When families cannot guarantee care for people with Dementia, admission to a facility may avoid social isolation and prevent abuse.	○	○	○
4- Provide a normal diet, while assessing the causes of dysphagia.	○	○	○
5- Non-pharmacological and pharmacological measures should be used together to manage the different behavioural and psychological symptoms of dementia.	○	○	○
6- Reporting the existence or suspicion of abuse is not a matter for nurses or elderly care workers, but for other professionals.	○	○	○
7- The management of extreme agitation, violence and aggressiveness must take place in a safe, low-stimulation environment, separate from other users of the service.	○	○	○
8- Specific drugs are the first option for treatment of psychological and behavioural disorders.	○	○	○
9- The application of mechanical restraints, type and date of application, reason, care provided and informed consent should be recorded in the Patient Medical Record.	○	○	○
10- Palliative care must include psychosocial, spiritual, cultural and family support aspects.	○	○	○
11- Conduct long-term physical activity programs to maintain the functional capacity of institutionalized dementia patients.	○	○	○
12- Use the oral route for fluid supply at the end of life, whenever possible.	○	○	○
13- Informing family and caregivers of the near death situation does not improve care in the last few days.	○	○	○
14- Inform the caregiver about the disease and its possible complications, and the social resources and support systems available.	○	○	○

	Yes	No	Don't know
15- Behaviour modification, programmed hygiene and induced micturition increase urinary incontinence in patients with dementia.	○	○	○
16- Identify who is the patient's representative to include him or her in decision-making and care planning.	○	○	○
17- Caregivers should be informed and trained to prevent the onset of behavioural and psychological symptoms of dementia.	○	○	○
18- Advise the person with dementia, to prepare the living will document in the early stages of the disease.	○	○	○
19- Provide comprehensive care to the caregiver, including counselling and emotional support.	○	○	○
20- Intervention programmes on activities of daily living do not reduce caregiver burden in the medium.	○	○	○
21- Record in the Patient Medical Record data on the form of onset, progression, and psychological and behavioural symptoms.	○	○	○
22- Care plans should address activities of daily living to maximize independent activity, maintain function, adapt and develop skills.	○	○	○
23- Use nasogastric tube or percutaneous gastrostomy in the patient with advanced dementia as a regular feeding route, if dysphagia.	○	○	○
